# Christianity Aggressive

**Published:** 1873-11

**Authors:** 


**Christianity Aggressive.**—The defensive armor of a shrinking or timid policy does not suit her. Hers is then aked majesty of truth; and with all the grandeur of age, but with none of its infirmities, has she come down to us, and gathered strength from the battles she has fought and the victories she has won in the many conflicts of many generations. With such a religion as this, there is nothing to hide. All should be above board; and the broadest light of day should be made freely to circulate through all her secresies. But secrets she has none. To her belong the frankness and simplicity of conscious greatness; and whether she grapple it with pride of philosophy, or stand in front opposition to the prejudices of the multitude, she does it upon her own strength, and spurns all the props and all the auxiliaries of superstition away from her.—Dr. Chalmers

